# Endovascular Treatment of Infrarenal Abdominal Aortic Aneurysm with Short and Angulated Neck in High-Risk Patient

**DOI:** 10.1155/2013/898024

**Published:** 2013-07-01

**Authors:** Stylianos Koutsias, Georgios Antoniou, Christos Karathanos, Vassileios Saleptsis, Konstantinos Stamoulis, Athanasios D. Giannoukas

**Affiliations:** ^1^Department of Vascular Surgery, University Hospital of Larissa, University of Thessaly Medical School, 41000 Larissa, Greece; ^2^Department of Anaesthesiology, University Hospital of Larissa, University of Thessaly Medical School, 41000 Larissa, Greece

## Abstract

Endovascular treatment of abdominal aortic aneurysms (AAA) is an established alternative to open repair. However lifelong surveillance is still required to monitor endograft function and signal the need for secondary interventions (Hobo and Buth 2006). Aortic morphology, especially related to the proximal neck, often complicates the procedure or increases the risk for late device-related complications (Hobo et al. 2007 and Chisci et al. 2009). The definition of a short and angulated neck is based on length (<15 mm), and angulation (>60°) (Hobo et al. 2007 and Chisci et al. 2009). A challenging neck also offers difficulties during open repairs (OR), necessitating extensive dissection with juxta- or suprarenal aortic cross-clamping. Patients with extensive aneurysmal disease typically have more comorbidities and may not tolerate extensive surgical trauma (Sarac et al. 2002). It is, therefore, unclear whether aneurysms with a challenging proximal neck should be offered EVAR or OR (Cox et al. 2006, Choke et al. 2006, Robbins et al. 2005, Sternbergh III et al. 2002, Dillavou et al. 2003, and Greenberg et al. 2003). In our case the insertion of a thoracic endograft followed by the placement of a bifurcated aortic endograft for the treatment of a very short and severely angulated neck proved to be feasible offering acceptable duration of aneurysm exclusion. This adds up to our armamentarium in the treatment of high-risk patients, and it should be considered in emergency cases when the fenestrated and branched endografts are not available.

## 1. Introduction

Endovascular treatment of abdominal aortic aneurysms (AAA) is an established alternative to open repair. However lifelong surveillance is still required to monitor endograft function and signal the need for secondary interventions [1].

Endovascular repair (EVAR) may not always be the best treatment option, as not all patients are eligible for EVAR owing to aortoiliac anatomy. Severe infrarenal aortic neck angulation is clearly associated with proximal type I endoleak, while its relationship with stent-graft migration is not clear [2]. Excluder, Zenith, and Talent stent grafts perform well in patients with severe neck angulation, with only a few differences among devices [2]. Aortic morphology, especially related to the proximal neck, often complicates the procedure or increases the risk for late device-related complications [2, 3]. The definition of a short and angulated neck is based on length (<15 mm) and angulation (>60°) [2, 3].

Fenestrated stent grafts crossing the orifices of the renal arteries have been developed to overcome insufficient neck lengths [4]. Its deployment in an angulated neck is considerably more risky than in a straight neck.

Dilatation of the proximal infrarenal aortic neck, which was found to be another predictor for endograft migration in an earlier EUROSTAR report [5], was also associated with severe neck angulation.

However, a challenging neck also offers difficulties during open repairs (OR), necessitating extensive dissection with juxta- or suprarenal aortic cross-clamping. Patients with extensive aneurysmal disease typically have more comorbidities and may not tolerate extensive surgical trauma [6]. It is, therefore, unclear whether aneurysms with a challenging proximal neck should be offered EVAR or OR [7–12].

In this report, a new method for better stent-graft fixation in a short and angulated aortic neck with the use of currently available devices is presented.

## 2. Case Report

**Figure 1:**
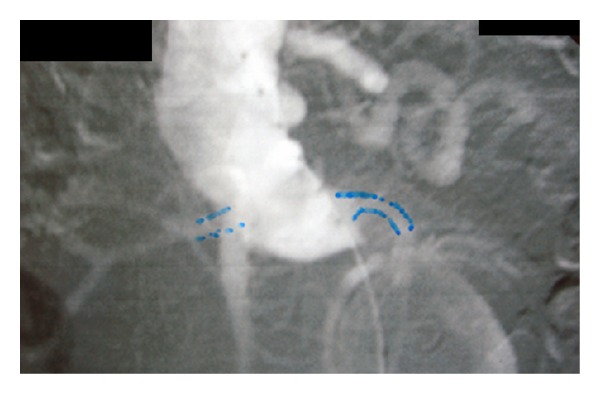
DSA arteriography that shows the short and angulated neck of the AAA.

**Figure 2:**
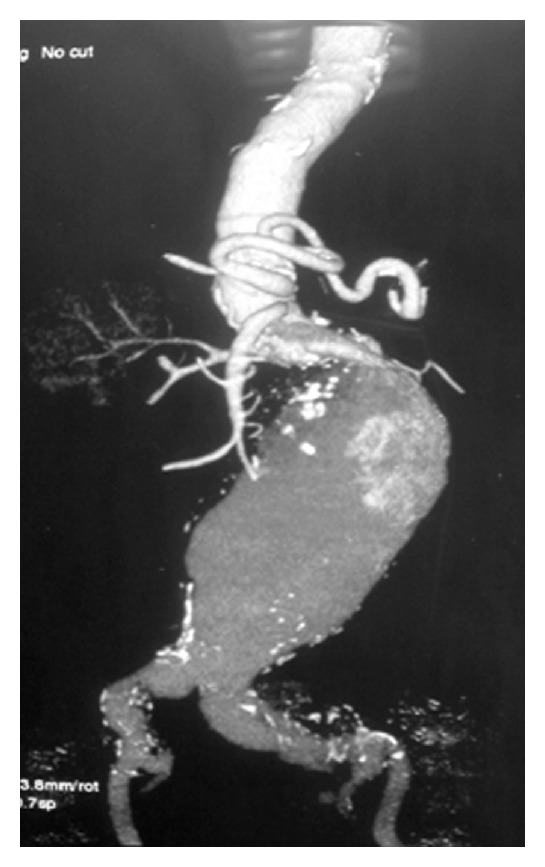
CT angiography of the anatomy of the AAA.

A 77-year-old male was admitted from the emergency unit with sudden onset of abdominal pain radiating to lumbar area. On CT scan an infrarenal AAA with maximum diameter of nine (9) cm was discovered. The patient was hypertensive with history of aortocoronary bypass 15 years ago. Shortly after that operation, he had subsequent sternum removal for infection. The coronary grafts were detected and occluded eleven (11) years ago on the occasion of a myocardial infarction that he had suffered. One year later, he had one more episode of myocardial infarction, but since then, he was moderately symptomatic on medical treatment. The patient was considered inoperable for his coronary disease. Anatomic characteristics of the infrarenal aortic neck were unfavourable due to its shortness (~8 mm) and angulation (>60°) (Figures 1 and 2).

 It was decided to treat him with EVAR, having his consent. The endovascular procedure was carried out in an operating room (OR) equipped with portable C-arm fluoroscopy device (Philips, Endura) and a radiolucent table.

**Figure 3:**
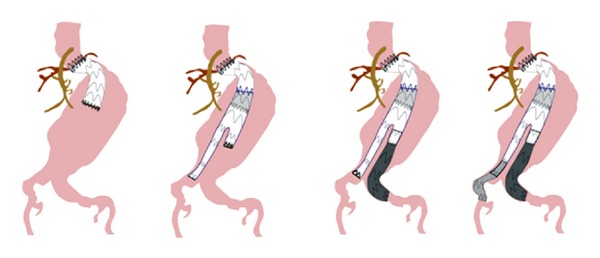
The sequence of the graft insertion.

**Figure 4:**
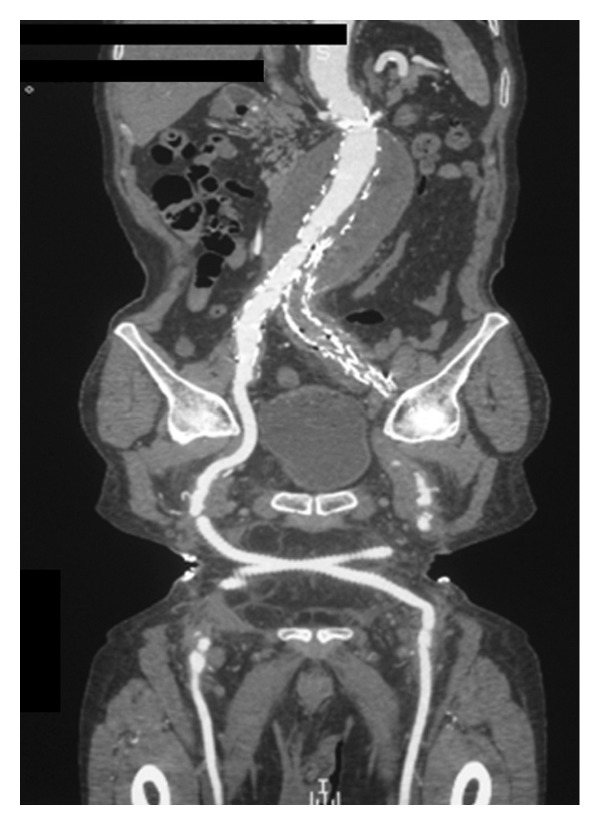
Complete exclusion of the aneurysm on CT angiography one month postoperatively.

**Figure 5:**
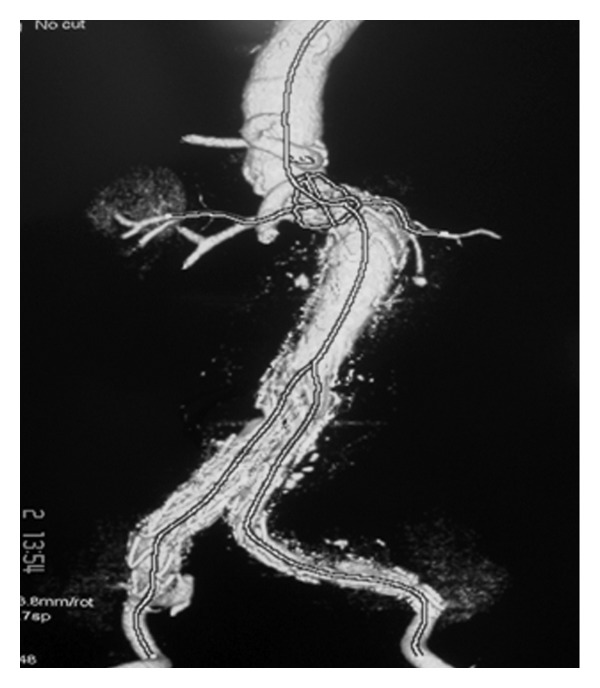
No endoleak was detected on CT angiography nine months postoperatively. The left limb of the graft is occluded, and the femorofemoral crossover bypass is patent.

 A 30 mm diameter, free flow thoracic tube endograft (Valiant, Medtronic Vascular, Santa Rosa, CA, USA) was delivered in the proximal neck. Consequently, a bifurcated Talent (Medtronic Vascular, Santa Rosa, CA, USA) device 32X18X155 was deployed inside the Valiant graft, with adequate overlapping. Two sequential iliac extensions were deployed into the left external iliac artery, and a contralateral limb was placed to the right common iliac artery (Figure 3). CT angiogram at first month documented intact 3-component stent graft, with no endoleak or migration and no increase in aneurysm sac (Figure 4) A month later the left limb was occluded causing intermittent claudication. Endovascular attempt to salvage the left limb of the graft was unsuccessful due to the tortuosity of the external iliac artery. A crossover fem-fem PTFE graft (8 mm) was placed with full restoration of the blood flow to the left lower extremity. Nine (9) months postoperatively, the patient underwent CT angiography that showed no endoleak and good functioning of the thigh-femoral graft (Figure 5). 

**Figure 6:**
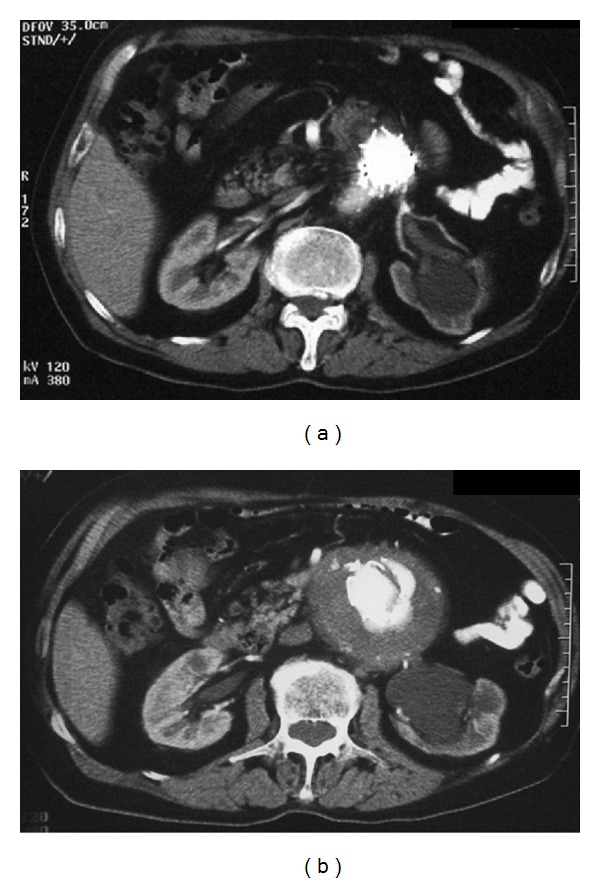
CT scanning one year after EVAR. (a) Migration of the endograft to the straight part of the neck. (b) Endoleak type I detected in the aneurysm.

 About a year from the EVAR the patient was admitted from the Emergency Unit with an episode of abdominal pain. On CT scan a type I endoleak was discovered along with mild graft migration (Figures 6(a) and 6(b)). However the abdominal pain was subsided with appropriate control of his hypertension, the patient remained haemodynamically stable, and he decided not to have any further intervention. Then he was discharged with the advice to be on close follow-up and meticulous management of his hypertension. 

He remained asymptomatic for another one year when he was admitted with abdominal pain, severe hypotension (systolic BP 60 mm Hg), and oliguria and CT scan showed rupture with contained large retroperitoneal hematoma. The patient was taken urgently to OR and subjected to open repair. The endograft was removed followed by the insertion of a tube 22 mm Dacron. He was transferred to ICU, and the next day he died.

## 3. Discussion

Conway et al. [13] reported that mortality caused by AAA rupture after a 3-year follow-up in 106 patients considered as high risk for open treatment was 36% in patients with aneurysms between 5.5 and 5.9 cm in diameter, 50% for aneurysms between 6.0 and 7.0 cm, and 55% for aneurysms greater than 7 cm in diameter.

 Nonintervention in patients with AAA and high surgical risk is only justified in those with an extremely short life expectancy, in whom the risk of death associated with the surgical procedure is higher than the risk of death caused by aneurysm rupture [14]. In our case the patient was undoubtedly of high risk (ASA IV or Goldman score), but his aneurysm was symptomatic indicating imminent rupture, and having his consent, we proceeded with endovascular repair avoiding OR. 

 Taking into consideration the study published by Zanchetta et al. [15] (“funnel technique for first-line endovascular treatment of an abdominal aortic aneurysm with an ectatic proximal neck”), we decided to amend the method applied by changing the sequence of endografts inserted. The use of Valiant thoracic endograft was decided on the basis that its free flow design has eight peaks instead of five and has the following properties: allows for the same radial force as Talent Thoracic Stent Graft with significantly reduced flare and distributes radial force across more points of contact with less force and stress per point. To meet the challenges imposed by our patient's aortic anatomy, we used a composite 3-component stent graft to satisfy the short and angulated aortic neck and optimize graft fixation as well as to ensure adequate sealing.

Primary laparoscopic proximal aortic banding [16] or a fenestrated endograft [16, 17] might also be performed to treat patients with a similar anatomy, but these procedures are, respectively, more invasive and time consuming. Fenestrated and branched endografts also permit the endovascular treatment of juxtarenal and pararenal aneurysms. However, these endografts are not available in emergency cases as the delivery time is about 2-3 months.

In our case the insertion of a thoracic endograft followed by the placement of a bifurcated aortic endograft for the treatment of a very short and severely angulated neck proved to be feasible offering acceptable duration of aneurysm exclusion. This adds up to our armamentarium in the treatment of high-risk patients, and it should be considered in emergency cases when the fenestrated and branched endografts are not available.
